# Regarding: research progress of femoral head necrosis in HIV-infected patients

**DOI:** 10.1080/07853890.2025.2484666

**Published:** 2025-03-28

**Authors:** Yanli Wang, Xiaoshu Zhou, Ying Wen

**Affiliations:** a Department of Infectious Diseases, The First Affiliated Hospital of China Medical University, Shenyang, China; b Department of Orthopedics, The First Affiliated Hospital of China Medical University, Shenyang, China

We appreciate the academic contribution of the recent publication of Yang et al. [1], who highlighted the clinical features of femoral head necrosis in HIV-infected patients. However, we would like to address three key issues.

First, an increased incidence of osteonecrosis was previously reported in HIV-positive populations, especially in the post-antiretroviral therapy (ART) era, which was only based on retrospective HIV-infected small sample case studies [2,3]. However, the incidence of osteonecrosis among the general population was mainly based on large-sample studies, such as being approximately 1.4–4.2/100,000 person-years (PYs) and increasing to 4.7/100,00 PYs among older adults. Notably, the incidence of osteonecrosis among HIV-positive patients was lower than that among systemic lupus erythematosus patients, severe acute respiratory syndrome (SARS) patients, and renal transplant patients. Therefore, a large sample of epidemic data is urgently required for HIV- positive and ART- experienced populations.

Second, the pathogenesis of osteonecrosis due to disrupted blood supply should be explained by a ‘multi-hit hypothesis.’ Corticosteroids, presenting as time - and dose - dependent risks of osteonecrosis, are commonly used in HIV-positive patients with severe *pneumocystis* pneumonia and immune reconstitution inflammatory syndrome (IRIS). However, many reported cases of osteonecrosis lack a history of exposure to corticosteroids and alcohol [2]. Therefore, other factors, including genetic polymorphisms, concomitant diseases, and infections, also increase individuals’ vulnerability. In the general population, several common comorbid conditions, such as connective tissue diseases, cancer, chronic pulmonary diseases, liver disease, and renal disease, are associated with an increased risk of osteonecrosis. Notably, concomitant infections, such as Herpes Zoster virus, cytomegalovirus, Brucella, and oral microbiome, could also be linked to the pathophysiology of osteonecrosis. To date, the effects of HIV and anti-HIV drugs on osteonecrosis have been inconclusive. HIV-infected patients with low baseline CD_4_^+^ T cell  counts  presented with increased  osteonecrosis after ART initiation and might have undergone prior AIDS-defining illnesses and the most robust T cell reconstitution. The majority of patients with osteonecrosis have obviously restored CD_4_^+^ T cell  counts post-ART [3]. Persistent activation and dysregulation of the innate immune system are major drivers of non-AIDS comorbidities during virally suppressed HIV infection. Therefore, apart from micro-embolism, inflammatory osteoimmunology plays a vital role in the pathogenesis of osteonecrosis [4], mainly involving macrophages, T/B cells and neutrophils, which share comparable circumstances with IRIS [5]. Although cases of IRIS-related osteonecrosis have not been previously reported, rheumatoid arthritis has been previously reported as a manifestation of IRIS [5]. This inspired us to explore the interaction of immune and skeletal system and targeting therapies for osteonecrosis.

Thirdly, HIV-positive patients were predisposed to developing osteonecrosis and osteoporosis, especially during the first 2 years of ART. Among HIV-infected patients, osteoporosis in patients with osteonecrosis was more frequent than in those without osteonecrosis. Apart from the imbalance of bone resorption/formation, the regulatory role of immune cells in skeletal micro-environment during bone-cell differentiation might impose on the development of osteonecrosis.

## Data Availability

Data sharing is not applicable to this article as no new data were created or analyzed in this study.

## References

[1] Yang Y, Ke F, Pan Z, et al. Research progress of femoral head necrosis in HIV-infected patients. Ann Med. 2025;57(1):2451185. doi: 10.1080/07853890.2025.2451185. 39847393 PMC11758795

[2] Scribner AN, Troia-Cancio PV, Cox BA, et al. Osteonecrosis in HIV: a case-control study. J Acquir Immune Defic Syndr. 2000;25(1):19–25. doi: 10.1097/00126334-200009010-00003. 11064500

[3] Lee SO, Lee JE, Lee S, et al. Osteonecrosis of the femoral head in Korean patients with human immunodeficiency virus infection. Infect Chemother. 2020;52(4):592–599. doi: 10.3947/ic.2020.52.4.592. 33263239 PMC7779977

[4] Ma M, Tan Z, Li W, et al. Osteoimmunology and osteonecrosis of the femoral head. Bone Joint Res. 2022;11(1):26–28. doi: 10.1302/2046-3758.111.BJR-2021-0467.R1. 35045723 PMC8801166

[5] Manzardo C, Guardo AC, Letang E, et al. Opportunistic infections and immune re-constitution inflammatory syndrome in HIV-1-infected adults in the combined antiretroviral therapy era: a comprehensive review. Expert Rev Anti Infect Ther. 2015;13(6):751–767. doi: 10.1586/14787210.2015.1029917. 25860288

